# A decade of Ecuador´s efforts to raise its health research output: a bibliometric analysis

**DOI:** 10.1080/16549716.2020.1855694

**Published:** 2020-12-27

**Authors:** Ivan Sisa, Andrea Abad, Isabel Espinosa, Isaac Martinez-Cornejo, Pablo Burbano-Santos

**Affiliations:** a School of Medicine, College of Health Sciences, Universidad San Francisco de Quito USFQ, Quito, Ecuador; b Science & Health Research Group, Quito, Ecuador; c Biomedical Research Institute August Pi Sunyer (IDIBAPS), Barcelona, España

**Keywords:** National health research system, health research policy, policy analysis, biomedical research, bibliometric analysis, Ecuador

## Abstract

**Background**: Over the past decade, the political movement called ‘Revolución Ciudadana’ implemented a variety of policies and interventions (P&I) in Ecuador to improve higher education and strengthen local research capacity. We refer specifically to the ‘Mandato 14’ and the Higher Education Law (LOES, Spanish acronym) launched in 2008 and 2010, respectively.

**Objective**: To assess the impact of these P&I (Mandato 14/LOES) on the production of health sciences-related articles (HSRA), and the relationship of these HSRA with the country’s health priorities.

**Methods**: A Scopus search was performed to retrieve HSRA published from 1999 to 2017. Bivariate analysis was used to assess variation between the period I (1999–2008) and period II (2009–2017). Further, we examined the association between the top 10 causes of mortality and the total HSRA output.

**Results**: The final study sample consisted of 2784 articles. After 2008, Ecuadorian production of HSRA increased steadily from 671 to 2133 publications (p<.001). Overall (1999–2017), the most common study design was cross-sectional (32.3%), the primary research focus was in the clinical-surgical area (49.3%), and the academic institutions were the primary drivers of scientific production during period II (56.9% vs. 29.5%, p<.001). Further, we found a decrease in the production of randomized controlled trials (6.7% vs. 1.8%, p<.001). Only 9% of research production involved the primary causes of mortality, and the proportion has remained unchanged over time (8.2% vs. 9.3%, p>.05).

**Conclusions**: Ecuadorian HSRA output increased significantly after 2008. This larger volume of scientific output could be the result to the Mandato 14/LOES implemented in the last decade. However, a low percentage of HSRA are dedicated to addressing the country’s health priorities. Proper planning, execution and monitoring of national health research agendas would reduce the mismatch between health burden and the HSRA output in Ecuador and other low-and middle-income countries.

## Background

The advancement of the practice of modern medicine is based on the production of biomedical research. As such, enormous resources are invested every year to execute this complex enterprise. For example, in 2010, ~ US$240 billion was invested in health research and development (R&D) globally [1]. It is essential that such research be of high quality and impactful because ultimately, policy makers, research stakeholders, health-care providers, and clinicians utilize it in order to improve population health and health equity [2]. Therefore, there is an increasing demand for evaluation of medical research, especially for research funders who expect that the research they fund will fulfill its anticipated aims and have an impact in terms of tangible returns [3].

Over recent decades, despite political turmoil and economic crisis, research in the Latin America and the Caribbean (LAC) region has advanced considerably due to widespread efforts to improve higher education and research capacity [4]. Several countries in the region have implemented national quality assurance and evaluation mechanisms in hopes of improving local higher education systems [5]. Thus, the LAC region has increased its overall expenditure on R&D from 0.57% to 0.8% of the gross domestic product (GDP) [6].

Likewise, Ecuador, an Andean country located in northwestern South America, implemented several policies and interventions (P&I) in the healthcare and higher education sectors under President Rafael Correa´s administration (2007–2017) that were aimed at strengthening local research capacities and improving population health [7]. For instance, in the health-care sector, Ecuador’s Ministry of Public Health implemented a new state-centered model for healthcare delivery. This model (MAIS-FCI) used the renew primary health-care strategy as its conceptual framework to organize and operate the Ecuadorian health-care system [8]. The MAIS-FCI model is based on providing health-care services by strengthening human resources and research activities. In that sense, MAIS-FCI encourages research efforts that tackle priority local diseases burden and their determinants [9]. In the same manner, in the higher education sector, the government launched two landmark initiatives: in 2008 the ‘Mandato 14’ and in 2010 the Higher Education Law (LOES, Spanish acronym) [10]. The ‘Mandato 14’ released by the National Assembly performed a national assessment of the Ecuadorian universities [11]. As a result of this evaluation, local universities were ranked into 5 categories and after 2 years, 14 universities in the lowest category were permanently closed [12]. On the other hand, LOES was enacted to promote research through the implementation of regulations and programs in Ecuador [7]: (i) several government offices were created to oversee university administration, faculty qualifications, and expectations for universities’ research output; (ii) the LOES mandated that all faculty members to have at least a master’s degree to teach at the university level; (iii) faculty without a doctoral degree were given a 7-year deadline to obtain one; and (iv) the law also addressed faculty employment contracts. Before the LOES, a regular faculty workweek was of 30 hr, so having a second job was a common practice. Currently, faculty members are expected to work full time in only one institution and to spend the additional 10 hr of the regular week in research-related activities. Additionally, in order to support faculty and universities in building research capacity as required by LOES, two programs were implemented. The first consisted in a study-abroad grant program for Ecuadorian students and faculty members to pursue graduate degrees in foreign universities all around the globe. The second program, called ‘Prometeo-Viejos Sabios’ program, was designed to bring foreign research expertise into the country. Prometeos scholars were placed in an Ecuadorian university for between three months and a year. Their main role was to stimulate faculty research and technology transfer [7,10,12]. Under the LOES, all research and teaching efforts conducted in the country had to be aligned with the country´s social, economic, and health needs [10]. These P&I were launched via a post-neoliberal political movement called the ‘Citizens’ Revolution’ (Revolución Ciudadana) [13]. Yet, the impact of these P&I, in the health sciences sector, especially Mandato 14 and LOES, has not been systematically examined. A previous study examined the characteristics and trends of health sciences-related articles (HSRA) published in Ecuador [14]. This study found that 625 HSRA were published from 1999 to 2009, primarily in the clinical and surgical areas (60%). Further, only 7.2% of the total production was related to the primary causes of Ecuadorian mortality [14]. The aims of the present study are to provide an update in data on HSRA as well as to assess the impact of the Revolución Ciudadana´s P&I (M14 and LOES) on the scientific output of health sciences in the country and its relationship with the primary causes of mortality, as a proxy for research prioritization within the Ecuadorian health-care system. To fill this gap, we conducted a bibliometric analysis of HSRA published from 1999 to 2017 in Ecuador.

## Methods

This is a bibliometric study used the Scopus database to identify HSRA published in Ecuador during the period 1999 to 2017. This citation index database was chosen because of (i) higher coverage of journals than PubMed and Web of Science (WoS) [15]; (ii) the greater correlation (R2 ~ .99) with WoS for citation analysis [16]; and (iii) its capacity to export, save, or e-mail the search results.

### Search strategy and selection criteria

We restricted our searching to articles and review documents published in any language. We searched in Scopus using the following equation:

AFFILCOUNTRY (*ecuador*) AND PUBYEAR > *1998* AND PUBYEAR < *2018* AND (LIMIT-TO (SUBJAREA, “*MEDI*”) OR LIMIT-TO (SUBJAREA, “*ENVI*”) OR LIMIT-TO (SUBJAREA, “*BIOC*”) OR LIMIT-TO (SUBJAREA, “*IMMU*”) OR LIMIT-TO (SUBJAREA, “*NEUR*”) OR LIMIT-TO (SUBJAREA, “*PHAR*”) OR LIMIT-TO (SUBJAREA, “*VETE*”) OR LIMIT-TO (SUBJAREA, “*NURS*”) OR LIMIT-TO (SUBJAREA, “*MULT*”) OR LIMIT-TO (SUBJAREA, “*HEAL*”) OR LIMIT-TO (SUBJAREA, “*DENT*”)) AND (LIMIT-TO (DOCTYPE, “*ar*”) OR LIMIT-TO (DOCTYPE, “*re*”)).

Four members of the research team conducted all screening. Identified records were screened by title and abstract before full-text screening of the publications that were identified for final data extraction. Publications were eligible if they satisfied the following criteria: (i) related to the health sciences, (ii) reporting studies conducted/executed in Ecuador, (iii) published during the period of 1999 to 2017, and (iv) containing all the pre-specified study variables. We excluded studies of non-human subjects, duplicate papers, and references for documents for which it was not possible to obtain a full article.

### Data extraction

We developed and tested one data extraction form to collect and organize additional data about (i) ‘institutional affiliation’; (ii) ‘type of study design’; (iii) ‘research focus’ in basic science, public health, clinical/surgical, and translational research (definitions used for each research area are provided in the supplementary online material in Supplementary Table 1); and (iv) relationship with the top 10 causes of mortality in Ecuador, categorized as two standardized rankings (Supplementary Table 2) of the 10 causes of mortality derived from the annual rates reported for the Ecuadorian National Institute of Statistics and Census (INEC). Finally, identified publications resulting from the ‘Prometeo program’ were included as a ‘yes’ or ‘no’ variable. To ascertain these contributions, we assessed authorship and respective affiliations as well as the acknowledgment section of each manuscript to verify any funding support disclosing connecting the Ecuadorian government through its Secretariat of Higher Education. In order to assure consistency and accuracy of extracted data, the reviewers were trained and used standardized definitions. In addition, the first (IS) and last author (PB) proofread the extracted data in a random fashion. Discrepancies in data screening were discussed and resolved by consensus between the first and last author. Reasons for exclusion were identified and documented.

### Statistical analysis

Descriptive statistics were used to summarize the baseline characteristics of the study publications included in the study. Continuous variables were reported as median and interquartile range (IQR) due to skewness and categorical variables were reported as frequencies and percentages. We defined a pre-policy intervention period before 2008. The rationale to set this cut-off point was that the first *Revolución Ciudadana* initiative in the sector started in 2007 with the declaration of ‘A State of Emergency for Education’ [7]. Parametric (chi-squared) and non-parametric (Wilcoxon Rank Sum and Fisher’s exact test) tests were used to assess variation between the periods 1999–2008 (period I) and 2009–2017 (period II). In addition, we performed a subgroup analysis of the publications related to the top 10 causes of mortality based on INEC’s annual reports and specific research categories adapted from Serrano et al. (Supplementary Table 3) [17]. Results with statistical significance were those with a two-tailed p-value <0.05. All statistical analyses were conducted using R for Mac, V. 3.2.2.

## Results

**Figure 1.**
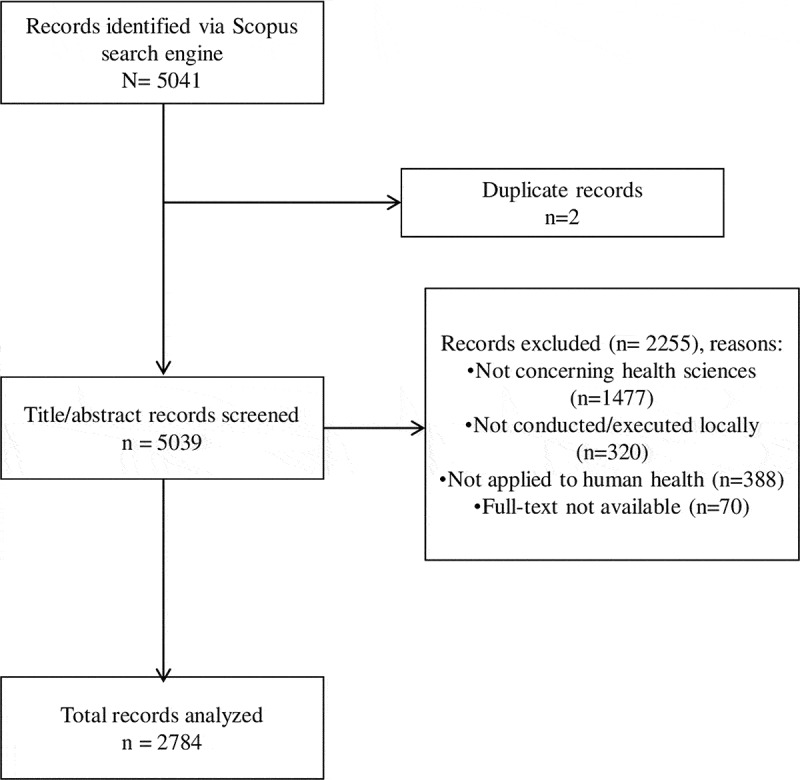
STROBE flowchart

Our search strategy identified 5041 HSRA in Scopus. After exclusion of duplicates (2 publications, 0.04%) and publications that did not satisfy the inclusion criteria (2255 publications, 44.7%), a total of 2748 publications were retrieved for the final review and analysis (Figure 1).

### Time trend of HSRA production in Ecuador

**Figure 2.**
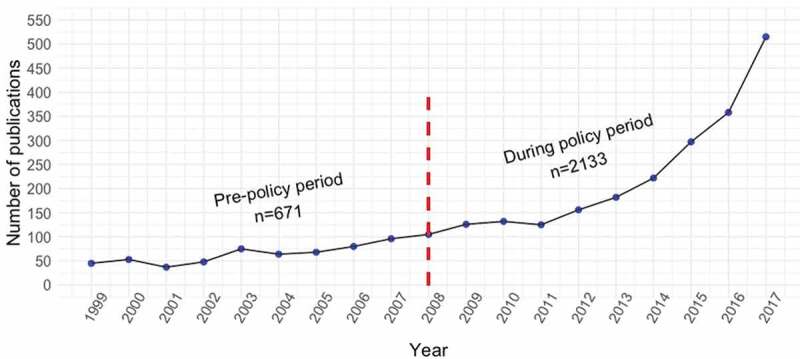
Ecuadorian trend of health science related-publications from 1999 to 2017 The period cut-off point is indicated by the dashed-red line

The total HSRA production increased by ~215% between period I (1999–2008) and period II (2009–2017), from 671 to 2133 publications (Supplementary Table 4). Overall, there has been a steady increase in the number of HSRA publications in Ecuador across the 18 years analyzed (Figure 2), and during period II, the production of HSRA increased significantly compared to the period I, to 182 (132–297) from 66 (49–79) publications, respectively, (p < .001). During period II, the principal document type was original papers (89.6%), English was the predominant language (76.6%) of publication, and the most common study design was cross-sectional (32.3%) as shown in Table 1. The largest increases in study design types published were in ecological, case-control, and cross-sectional studies, by 6200%, 800%, and 461%, respectively (Supplementary Table 4). Yet, we were surprised to find a decrease in the production of randomized controlled trials (RCTs) in the country from 6.7% of the total (n = 45) to 1.8% (n = 38), p < .001 (Table 1).

**Table 1.** Study characteristics of Ecuadorian health sciences-related publications, 1999–2017

Characteristic	0verall period (1999–2017) *n* = 2784	Period I (1999–2008) *n* = 671	Period II (2009–2017) *n* = 2113	p-value^a^
Articles, median (IQR)	105 (66–169)	66 (49–79)	182 (132–297)	<0.001
Language, n(%)				<0.01
English	2133 (76.6)	526 (78.5)	1607 (76)	
Spanish	546 (19.6)	133 (19.8)	413 (19.5)	
Other	105 (3.8)	12 (1.8)	93 (4.4)	
Publication type, n(%)				<0.001
Original paper	2496 (89.6)	574 (85.5)	1922 (90.9)	
Review	288 (10.3)	97 (14.4)	191 (9)	
Study design, n(%)				<0.001
Ecologic	128 (4.6)	2 (0.3)	126 (5.9)	
Cross-sectional	899 (32.3)	136 (20.3)	763 (36.1)	
Case-control	90 (3.2)	9 (1.3)	81 (3.8)	
Cohort	99 (3.5)	32 (4.8)	67 (3.2)	
Randomized controlled trial	83 (2.9)	45 (6.7)	38 (1.8)	
Meta-analysis	4 (0.1)	1 (0.1)	3 (0.1)	
Review	335 (12)	86 (12.8)	249 (11.8)	
Other	1146 (41.2)	360 (53.6)	786 (37.2)	
Institution affiliation, n(%)				<0.001
Private University	681 (24.5)	106 (15.8)	575 (27.2)	
Public University	523 (18.8)	66 (9.8)	457 (21.6)	
Private and Public University	71 (2.5)	6 (0.9)	65 (3.1)	
Private Hospital	250 (8.9)	118 (17.6)	132 (6.2)	
Public Hospital^b^	233 (8.4)	96 (14.3)	137 (6.5)	
Hybrid Hospital (SOLCA)	39 (1.4)	10 (1.5)	29 (1.4)	
Private and Public Hospital	49 (1.8)	25 (3.7)	24 (1.3)	
University and Hospital	350 (12.6)	81 (12.1)	269 (12.7)	
Industry	65 (2.3)	10 (1.5)	55 (2.6)	
Other	523 (18.8)	153 (22.8)	370 (17.5)	
Research focus, n(%)				<0.001
Basic science	606 (21.8)	136 (20.3)	470 (22.2)	
Clinical/surgical	1372 (49.3)	378 (56.3)	994 (47)	
Public health	783 (28.1)	156 (23.2)	627 (29.7)	
Translational	23 (0.8)	1 (0.1)	22 (1)	
Prometeo program, n(%)				
Yes	60 (2.1)	0	60 (2.8)	<0.001
Mortality, n(%)				
Yes	252 (9)	55 (8.2)	197 (9.3)	0.37

IQR = interquartile range; SOLCA = Sociedad de Lucha Contra el Cancer (Cancer Fighting Society).

^a^Continuous variables were analyzed by using Wilcoxon Rank Sum test; all categorical data were analyzed through Chi-squared or Fisher’s exact tests as appropriate.

^b^This category encompasses all members of the Ecuadorian public health system, including Ministry of Public Health (MSP), Ecuadorian Social Security Institute (IESS), Army Social Security System (ISSFA) and Police Social Security System (ISPOL).

### Main actors responsible for the HSRA production in Ecuador

Overall (1999 to 2017), private universities were the main actor with the highest number of HSRA publications (24.5%); public university and other institutions ranked second (18.8%), followed by the association between the university and hospital institutions (12.6%) (Table 1). However, hospital institutions (private, public, and hybrid) were the primary drivers of HSRA publications during the pre-policy period (1999–2008), compared to the period from 2009 to 2017 (37.1% vs. 15.2%, respectively; p < .001) (Supplementary Table 5). During the *Revolución Ciudadana*, public and private academic institutions took the lead of the production of HSRA publications and showed the largest increase in percent change, by 466% (Supplementary Table 5).

### Primary research focus of HSRA publications in Ecuador

**Figure 3.**
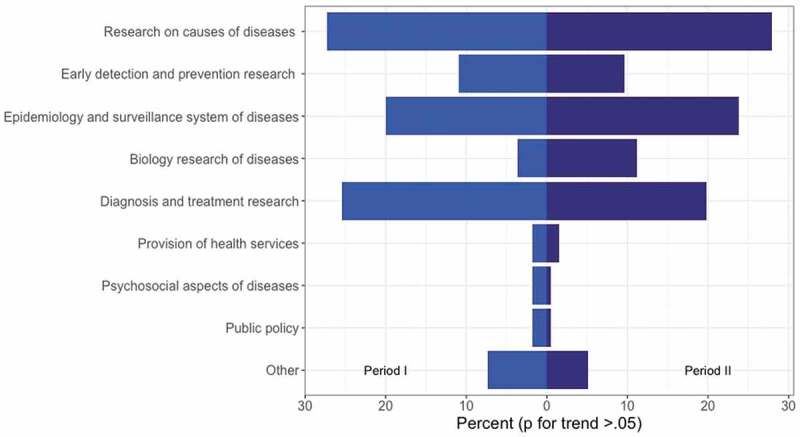
Distribution of Ecuadorian publications related to the top ten causes of mortality according to specific research lines

Across the 18 years analyzed, clinical and surgical topics were the primary focus of research (49.3%) for Ecuadorian healthcare researchers (Table 1). This finding is consistent with the subgroup analysis showing that research on causes of diseases; quantification of the disease burden and surveillance; and diagnosis and treatment research predominated across the analyzed period (Figure 3), without statistical difference across both time periods (p > .05). Yet, comparing both periods, the research production in the areas of basic science, public health, and translational research increased significantly during period II (2009–2017) compared to clinical/surgical publications (Table 1). The largest increase was observed in the area of translational research (Supplementary Table 4). In comparing scientific production of public and private academic we found that private universities conducted more research using cross-sectional, cohort, and RCT designs than public universities during the post-policy period. Among academic institutions, public universities performed more research using other experimental designs (40.7% vs. 31.8%, p < .05), which could explain their greater focus on basic science research compared to private institutions. Private universities performed more research in the area of clinical and surgical areas (41.2% vs. 33%, p < 0.05), but there was no difference in the production of HSRA related to the public health area (Supplementary Table 6).

### Impact of government policies on the country’s health priorities

The proportion of HSRA publications related to the top 10 causes of mortality increased from 8.2% in the period I to 9.3% in the period II, but this increment did not reach statistical significance between the analyzed periods (p = 0.37) (Table 1). Despite the overall trend of no difference, there have been some signs of progress for diseases that were present in both periods. For example, diabetes, ischemic heart disease, and influenza & pneumonia showed increments in HSRA publications that addressed those topics (Table 2). Among academic institutions, private universities were more likely to publish research on the main causes of mortality in the country than public universities during period: II, 6.6% vs. 10.3%, p < .05 (Supplementary Table 6).

**Table 2.** Ecuadorian HSRA related with the top 10 causes of mortality according to the INEC, 1999–2017

Period I (1999–2008) *n* = 55		Period II (2009–2017) *n* = 197	
Disease	n (%)	Disease	n (%)
Diabetes	10 (18.2)	Diabetes	49 (24.9)
Ischemic heart disease	3 (5.4)	Ischemic heart disease	19 (9.6)
Hypertension	6 (10.9)	Hypertension	23 (11.7)
Cerebrovascular disease	21 (38.2)	Cerebrovascular disease	53 (26.9)
Influenza & pneumonia	2 (3.6)	Influenza & pneumonia	28 (14.2)
Road injuries	0	Road injuries	2 (1)
Heart failure	0	Chronic liver disease (Cirrhosis)	5 (2.5)
Diseases related to the prenatal period	14 (25.4)	Urinary tract disease	4 (2)
Interpersonal violence	0	Lower respiratory tract disease	12 (6.1)
Other causes	0	Stomach cancer	2 (1)

HSRA = health sciences-related articles; INEC = national institute of statistics and census.

### ‘Prometeo-Viejos Sabios’ program and HSRA publications in Ecuador

We found 60 HSRA (2009–2017) publications that mentioned the Prometeo program either in the authorship affiliation or in the acknowledgements section. However, a further analysis of these publications showed that although were conducted locally, 13 of the 60 publications addressed topics unrelated to Ecuador or used data from other countries (Supplementary Table 7). Of the remaining Prometeo HSRA publications (n = 43) the largest proportion (44.7%) came from the Central University of Ecuador (Supplementary Figure 1). 23.4% of these publications were produced in collaboration with Japan, followed by Venezuela (19.1%) (Supplementary Figure 2).

## Discussion

These results highlight the potential impact of the *Revoluación Ciudadana´s* P&I on the production of HSRA publications in Ecuador from 2007 to 2017. The primary findings are as follows. First, the P&I (M14 and LOES) implemented in the Ecuadorian higher education system may have resulted in an increased volume of HSRA publications. Second, academic institutions were the primary drivers of HSRA publications in the country. Third, most of the HSRA output consists of observational studies (55.6%). Fourth, the primary research focus is in the clinical-surgical area. Fifth, a low percentage of the overall HRSA production was dedicated to addressing the country’s health priorities, specifically, the primary causes of mortality.

### Comparison with other studies

Ecuador significantly increased its production of HSRA publications across the entire period, and especially over the past decade, from 671 to 2133 publications. This growth could be the result of M14 and LOES, which catalyzed improvements in the qualification and accreditation processes of local higher education institutions (HEI), increased the investment of substantial funds for the development of a critical mass of scientists, provided for more government funding of research projects, and enhanced international collaboration [7,10] (Supplementary Figure 3). Countries with historically low scientific productivity such as Ecuador tend to emphasize research in health sciences after the implementation of government interventions aimed at increasing research production [18]. For example, a bibliometric analysis using Scopus found that from 2006 to 2015, Ecuador published 6548 scientific articles, with agriculture/biological sciences (28.8%) and medicine (27.8%) being the most popular areas [19]. However, this trend could be due to the availability of more data in the form of national datasets, hospital-related databases, and data collected as part of small survey studies.

Before the *Revolución Ciudadana’s* P&I, the main drivers of HSRA publication were private universities and hospitals [14]. We believe that two relevant milestones changed this trend. First, in 2010, LOES was passed with the goal of overseeing the higher education system in Ecuador [7,8,11,12]. Under this law, a new accrediting body for HEI (CEAACES, Spanish acronym) was created to standardize and raise the quality of education [7,19]. In 2012, this new agency implemented an institutional ranking mechanism to ensure compliance, at the same time putting greater emphasis on research endeavors. In order to be ranked as a teaching-research university 70% of the faculty body was required to have a PhD degree [7,19]. Second, in 2014, the Ecuadorian Ministry of Public Health launched a technical norm in order to regulate and qualify public and private health-care institutions as teaching assistance units or university hospitals. In both scenarios, the affiliation with an HEI was a requirement [20]. As a result, Ecuadorian HEIs took a major role in the generation of health sciences research and also facilitated the academic affiliation of hospital mentors with an HEI.

Observational studies were the publication study designs most commonly carried out and reported produced during the past decade, especially cross-sectional (36.1%, n = 763), review (11.8%, n = 249), and ecological (5.9%, n = 126) studies. Methodologically speaking, these study designs are useful for establishing preliminary evidence of a causal relationship. In contrast, there were few studies of a higher level of evidence to inform health policy-making such as cohort, RCTs and meta-analysis studies: 3.5% (n = 99), 2.9% (n = 83) and 0.1% (n = 4), respectively. We were surprised to find a decreasing trend for cohort and RCTs studies, from 4.8% to 3.2% and 6.7% to 1.8%, respectively (p < .001). This shift could be explained by the fact that analytical studies demand more financial, time, and logistical resources or by a lack of skilled personnel, or by the pressure exerted by the LOES institutional ranking. Ecuadorian researchers may have chosen to perform quick, relatively easy, and inexpensive studies in order to satisfy the new institutional ranking mechanism. Other factors that could explain these findings are ethical and regulatory system obstacles [21]. In Ecuador, following approval by an accredited university-or hospital-affiliated IRB, health-related studies must pass a second evaluation process by the Ministry of Public Health in order to conduct either observational studies with biological samples or RCTs. Further, minimal risk studies (e.g. collection of blood samples by venipuncture) by local regulations are required to be assessed as a full board study. These additional requirements lengthen the research process and discourage researchers and sponsors.

Of the 2784 HSRA included in this study, 1372 (49.3%) of them were related to topics in the clinical/surgical area compared to other areas such as public health (28.1%, n = 783). Further, only 252 (9%) HSRA of the overall production addressed the top 10 causes of mortality in the country, and this proportion remained unchanged across both analyzed periods. This mismatch between the HSRA output and disease burden has been previously reported elsewhere [14,22–24] and could be explained by several factors, including: i) a persistent biomedical paradigm that systemically rewards diagnostic and treatment services, despite the present of the MAIS-FCI model and the constitutional emphasis on the provision of promotion and preventative health-care services, to the detriment of population and community health-care practice [8,13,22]; ii) a failure of policy implementation, perhaps due to inadequate definition of goals, lack of communication between researchers and policy-makers, or poor monitoring of national research priorities [24,25]; iii) a deficient role of the Ministry of Public Health in supporting an articulate and vibrant national health research system driven by strong country priorities [26]; iv) lack of funding dedicated to tackling main causes of mortality in the country; and v) lack of biomedical and public health faculty and researchers. For example, in 2014, Ecuador had 11,410 qualified researchers; however, only 11.5% of them worked in the medical sciences, compared to 17.6% and 14.6% in neighboring Colombia and Peru, respectively [27].

### Strengths and limitations of the study

Our investigation has several strengths. We included lab-based studies in our analysis and thus were able to assess certain diseases more likely to be addressed by these types of studies, such as cancer. Potential limitations should also be considered. Although we used a comprehensive database (Scopus) to retrieve Ecuadorian HSRA, otherwise eligible publications not indexed in Scopus (especially in Spanish-language journals) may have been omitted. However, due to the extensive period analyzed, we anticipated that the effect of any missed publications would be minimal. We did not measure agreement among reviewers; thus, the possibility of misclassification cannot be ruled out. This study did not assess other dimensions or indices to identify and evaluate the impact of research such as morbidity or disability-adjusted life years (DALYs) [24], research capacity-building, informing decision-making, health benefits, and economic and social benefits, since the data available to us did not provide the information needed to incorporate these complex indicators [28]. We cannot claim causality of the implemented P&I (M14 and LOES) despite the evident increased in HSRA over time. Future studies could use another approach, such as interrupted time series analysis, which allows for a robust assessment of an intervention effect using longitudinal data [29]. In addition, due to issues with completeness and quality of mortality data in Ecuador especially during the first period (1999–2008) of analysis, we cannot exclude the possibility of outcome misclassification bias [30].

### Health systems research implications

During the decade of the *Revolución Ciudadana* movement, Ecuador benefitted from the largest increase in revenues in contemporary history because of boom in oil, its most important export, and a concomitant growth in GDP, which grew from US$51 billion in 2007 to US$94.47 billion in 2013 [31]. This increase translated to a higher investment in education and health; the portion of GDP allocated to education grew from 2.5% in 2006 to 4.6% in 2014, while the health sector received a significant increase in funding from less than of 2% of GDP in 2004 to 9% of in 2015–around US$5 billion/year [32,33]. Thus, it appears that funding resources during the *Revolución Ciudadana* were not a constraint, as is commonly reported in Low-and Middle-Income Countries (LMICs) [25,34]. The experience suggests several lessons for other LMICs in similar scenarios, in order to avoid outcomes that are mismatched with citizens’ priorities. First, build a strong governance and management body anchored to the Ministry of Health or an independent government agency that is able to set, communicate, and implement the country’s health research agenda across all key stakeholders [35]. Second, define proper mechanisms for monitoring and evaluation of the national health research system [36]. Third, foster the development of interdisciplinary research teams devoted to tackling complex local health issues using novel approaches such as implementation science and comparative effectiveness research [37]. Fourth, incorporate new information and communication technologies to capture information systematically and in real time [38]. Fifth, assure sustainable funding beyond specific project life cycle [36].

## Conclusions

In summary, our study showed that Ecuadorian HSRA increased significantly after 2008. This larger volume of scientific output could be the result of the *Revolución Ciudadana’s* P&I (M14 and LOES) that were implemented in the past decade. Most of the health sciences research output consisted of observational studies and the primary research focus was in the clinical-surgical area. Despite these advances, a low percentage of HRSA have been dedicated to addressing the country’s health priorities, and the proportion has remained unchanged over time. Proper planning, execution, and monitoring of national health research agendas would reduce the mismatch between health burden and the HSRA output in Ecuador and other LMICs.

## Supplementary Material

Supplemental Material

## Paper context

Local research agendas poorly cover the country’s health priorities in developing countries. One reason for that phenomenon has been attributed to the scarcity of funding. After a decade of policies and interventions, Ecuador increased its health research output significantly in a context of no funding constraints; however, most of it was not related to the top 10 causes of mortality. Besides funding, proper planning, execution, and monitoring of national research agendas would reduce this mismatch.
