# Supplementary material for: A decade of Ecuador´s efforts to raise its health research output: a bibliometric analysis
Source: Glob Health Action. 2020 Dec 27;14(1):1855694. doi: 10.1080/16549716.2020.1855694 (PMC7782667; doi:10.1080/16549716.2020.1855694)
Supplement: Supplemental Material [file ZGHA_A_1855694_SM9866.docx]

**APPENDIX MATERIAL**

**A decade of Ecuador´s efforts to raise its health research output: A bibliometric analysis**

**Table of Contents**

**TABLES**…………………………………………………………………………………….3

**Table S1.** Definitions used to classify HSRA publications retrieved from Scopus, 1999-2017………………………………………………………………………………………….3

**Table S2.** Ten principal causes of mortality based on the INEC for the analyzed periods of time…………………………………………………………………………………………..5

**Table S3.** Definitions used to classify HSRA publications related to the top ten causes of mortality based on specific research lines, 1999-2017**……………………………………................................................................................**6

**Table S4.** Percent change between period I and period II of baseline characteristics associated with Ecuadorian HSRA publications, 1999-2017……………………………….7

**Table S5.** Ecuadorian health sciences-related publications by institutional affiliation, 1999-2017………………………………………………………………………………….............8

**Table S6.** Ecuadorian health sciences-related publications by public vs. private universities, 1999-2017………………………………………………………………………………………….9

**Table S7.** Prometeo-related publications published using data not related to Ecuador….............................................................................................................................10

**FIGURES**………………………………………………………………………………….12

**Figure S1.** Health science publications related to the Prometeo program according to institution affiliation………………………………………………………………………..12

**Figure S2.** Health science publications related to the Prometeo program by the country affiliation of the grant recipient…………………………………………………………….13

**Figure S3.** Timeline of the milestones achieved in Ecuadorian higher education system during “Revolución ciudadana”, 2007-2017……………………………………………….14

**Table S1. Definitions used to classified HSRA publications retrieved from Scopus, 1999-2017.**

| **Research focus** | **Definition** | **Taken from** |
| --- | --- | --- |
| Basic science research | This type of research encompasses familiar scientific disciplines such as biochemistry, microbiology, physiology, and pharmacology, and their interplay, and involves laboratory studies with cell cultures, animal studies or physiological experiments. Basic science also increasingly extends to behavioral and social sciences as well, which have no less profound relevance for medicine and health. Basic research can address clinical issues from a reductionist approach, including the discovery and analysis of single genes or genetic markers of diseases, or sequencing and manipulating genes. Typically, basic science research focuses on determining the causal mechanisms behind the functioning of the human body in health and illness, and utilizes hypothesis-driven experimental designs that can be specifically tested and revised | <https://www.aamc.org/initiatives/research/334422/basicscience.html> |
| Clinical/Surgical Research | Clinical research as a component of medical and health research intended to produce knowledge valuable for the understanding of human disease, preventing and treating illness, and promoting health. Clinical research involves interactions with patients, diagnostic clinical materials or data, or populations in any of the following areas: (1) disease mechanisms (etiopathogenesis); (2) bi-directional integrative (translational) research; (3) clinical knowledge, detection, diagnosis and natural history of disease; (4) therapeutic interventions including clinical trials of drugs, biologics, devices and instruments; (5) prevention (primary and secondary) and health promotion; (6) behavioral research; (7) health services research, including outcomes, and cost-effectiveness; (8) epidemiology; and (9) community-based trials. | <https://www.ncbi.nlm.nih.gov/books/NBK56985/> |
| Public health research | **Public Health Research** encompasses contributions from both the 'traditional' disciplines of public health, including hygiene, epidemiology, health education, environmental health, occupational health, health policy, hospital management, health economics, law and ethics as well as from the area of new health care fields including social science, communication science, eHealth and mHealth philosophy, health technology assessment, genetics research implications, population-mental health, gender and disparity issues, global and migration-related themes. | <http://www.jphres.org/> |
| Translational research | Translational research is the process of applying knowledge from basic biology and clinical trials to techniques and tools that address critical medical needs. Unlike applied sciences, translational research is specifically designed to improve health outcomes. It uses an integrated team of experts who are focused on translating useful information from laboratories to doctors’ offices and hospitals. It’s a “bench to bedside” bridge. In translational research, scientists who study biology and genetics partner with community members, engineers, pharmacists, bioinformaticists, policymakers and others. [Multidisciplinary collaborations](https://www.ucdavis.edu/one-health/collaborations) result in diagnostic devices, chemical agents, powerful algorithms, business enterprises and a culture of creating knowledge to answer complex medical questions. | <https://www.ucdavis.edu/one-health/translational-research/> |

**Table S2. Ten principal causes of mortality based on the INEC for the analyzed periods of time**

| **Diseases of period I (1999-2008)** | **Diseases of period II (2009-2017)** |
| --- | --- |
| Diabetes | Ischemic heart disease |
| Hypertension | Diabetes |
| Ischemic heart disease | Cerebrovascular disease |
| Heart failure | Hypertension |
| Cerebrovascular disease | Influenza & pneumonia |
| Influenza & pneumonia | Road injuries |
| Diseases related to the prenatal period | Chronic liver disease (Cirrhosis) |
| Road injuries | Urinary tract disease |
| Interpersonal violence | Lower respiratory tract disease |
| Other causes | Stomach cancer |

Note. INEC= Ecuadorian National Institute of Statistics and Census.

**Table S3. Definitions used to classify HSRA publications related to the top ten causes of mortality based on specific research lines, 1999-2017**

| **Research categories** | **Definitions** |
| --- | --- |
| Epidemiology and surveillance system of diseases | - Ongoing, systematic collection, analysis and interpretation of health data - Disease burden and mortality estimates |
| Research on causes of diseases | - Identification of potential causal relationships between exposure and diseases |
| Biology research of diseases (molecular and cellular mechanisms) | - The study of the fundamental processes of developing of communicable and non-communicable diseases - Lab-based research |
| Early detection and prevention research | - Developing and applying of methodologies to identify disease risk factors - Study of approaches to prevent the development of prevalent diseases |
| Diagnosis and treatment research | - Study of early detection (screening) of disease - Research on the provision of treatment modalities |
| Provision of health services | - Study of the provision of health care at a community and population level - Novel approaches to maximize health care services |
| Psychosocial aspects of diseases | - Study of behavioral determinants of disease |
| Public policy | - Study of the developing and implementation of public policy to tackle disease |
| Other | - Research lines not previously stated |

Note. This table has been built and adapted from reference 18

**Table S4. Percent change between period I and period II of baseline characteristics associated with Ecuadorian HSRA publications, 1999-2017**

| Characteristic | Associated frequency for HSRA publications | | Percent change between period I and period II^a^ |
| --- | --- | --- | --- |
|  | Period I (1999-2008) | Period II (2009-2017) |  |
| Number of HSRA publications | 671 | 2113 | 215 |
| Publication type |  |  |  |
| Original paper | 574 | 1922 | 235 |
| Review | 97 | 191 | 97 |
| Study design |  |  |  |
| Ecologic | 2 | 126 | 6200 |
| Cross-sectional | 136 | 763 | 461 |
| Case-control | 9 | 81 | 800 |
| Cohort | 32 | 67 | 109 |
| Randomized controlled trial | 45 | 38 | -16 |
| Meta-analysis | 1 | 3 | 200 |
| Review | 86 | 249 | 190 |
| Other | 360 | 786 | 118 |
| Institution affiliation |  |  |  |
| Private University | 106 | 575 | 442 |
| Public University | 66 | 457 | 592 |
| Private and Public University | 6 | 65 | 983 |
| Private Hospital | 118 | 132 | 12 |
| Public Hospital^b^ | 96 | 137 | 43 |
| Hybrid Hospital (SOLCA) | 10 | 29 | 190 |
| Private and Public Hospital | 25 | 24 | -4 |
| University and Hospital | 81 | 269 | 232 |
| Industry | 10 | 55 | 450 |
| Other | 153 | 370 | 142 |
| Research focus |  |  |  |
| Basic science | 136 | 470 | 246 |
| Clinical/surgical | 378 | 994 | 163 |
| Public health | 156 | 627 | 302 |
| Translational | 1 | 22 | 2100 |
| Mortality |  |  |  |
| Yes | 61 | 220 | 261 |

Note. HSRA= health sciences-related articles; SOLCA= Sociedad de Lucha Contra el Cancer (Cancer Fighting Society).

^a^ Percent changes correspond to ((Period II_estimates_ – Period I_estimates_)/ Period I_estimates_)100

^b^ This category encompasses all members of the Ecuadorian public health system, including Ministry of Public Health (MSP), Ecuadorian Social Security Institute (IESS), Army Social Security System (ISSFA) and Police Social Security System (ISPOL).

**Table S5. Ecuadorian health sciences-related publications by institutional affiliation, 1999-2017**

| Characteristic | 0verall period (1999-2017) | Period 1 (1999-2008) | Period 2 (2009-2017) | p-value |
| --- | --- | --- | --- | --- |
|  | n = 2784 | n = 671 | n= 2113 |  |
| Institution afillation, n(%) |  |  |  | <0.001 |
| Academia^§^ | 1275 (45.8) | 178 (26.5) | 1007 (51.9) |  |
| Hospital^§§^ | 571 (20.5) | 249 (37.1) | 322 (15.2) |  |
| Academia & Hospital | 350 (12.6) | 81 (12.1) | 269 (12.7) |  |
| Other^⌘^ | 588 (21.1) | 163 (24.3) | 425 (20.1) |  |

^§^ Encompasses public and privates universities.

^§§^ Encompasses public, private, and hybrid healthcare facilities.

^⌘^ Includes industry and other institutions.

**Table S6. Ecuadorian health sciences-related publications by public vs. private universities, 1999-2017**

| Characteristic | Period 1 (1999-2008) | |  | Period 2 (2009-2017) | |  |
| --- | --- | --- | --- | --- | --- | --- |
|  | Public n=66 | Private n=106 | p-value | Public n=457 | Private n=575 | p-value |
| Language, n(%) |  |  | <0.05 |  |  | <0.01 |
| English | 52 (78.8) | 99 (93.4) |  | 321 (70.2) | 489 (85) |  |
| Spanish | 12 (18.2) | 6 (5.7) |  | 117 (25.6) | 67 (11.6) |  |
| Other | 2 (3) | 1 (0.9) |  | 19 (4.2) | 19 (3.3) |  |
| Publication type, n(%) |  |  | 0.76 |  |  | 0.3 |
| Original paper | 58 (87.9) | 96 (90.6) |  | 416 (91) | 511 (88.9) |  |
| Review | 8 (12.1) | 10 (9.4) |  | 41 (8.9) | 64 (11.1) |  |
| Study design, n(%) |  |  | <0.05 |  |  | <0.05 |
| Ecologic | - | 1 (0.9) |  | 28 (6.1) | 44 (7.6) |  |
| Cross-sectional | 7 (10.6) | 30 (28.3) |  | 152 (33.3) | 227 (39.5) |  |
| Case-control | 1 (1.5) | 2 (1.9) |  | 19 (4.1) | 25 (4.3) |  |
| Cohort | 1 (1.5) | 7 (6.6) |  | 5 (1.1) | 15 (2.6) |  |
| Randomized clinical trial | 4 (6.1) | 7 (6.6) |  | 6 (1.3) | 12 (2.1) |  |
| Meta-analysis | - | - |  | 2 (0.2) | 1 (0.4) |  |
| Review | 8 (12.1) | 7 (6.6) |  | 59 (12.9) | 68 (11.8) |  |
| Other | 45 (68.2) | 52 (49) |  | 185 (40.7) | 183 (31.8) |  |
| Research focus, n(%) |  |  | <0.01 |  |  | <0.05 |
| Basic science | 25 (37.9) | 30 (28.3) |  | 154 (33.7) | 150 (26.1) |  |
| Clinical/surgical | 32 (48.5) | 37 (34.9) |  | 151 (33) | 237 (41.2) |  |
| Public health | 9 (13.6) | 39 (36.8) |  | 143 (31.3) | 181 (31.5) |  |
| Translational |  |  |  | 9 (1.9) | 7 (1.2) |  |
| Prometeo program, n(%) |  |  |  |  |  | <0.001 |
| Yes | - | - |  | 28 (6.1) | 4 (0.7) |  |
| Mortality, n(%) |  |  | 0.4 |  |  | <0.05 |
| Yes | 1 (1.5) | 5 (4.7) |  | 30 (6.6) | 59 (10.3) |  |

**Table S7. Prometeo-related publications published using unrelated data to Ecuador**

| **N** | **Title** | **Year** | **Prometo scholar associated** | **Ecuadorian host institution** |
| --- | --- | --- | --- | --- |
| 1 | Clinical and epidemiological features of leishmaniasis in northwestern-Argentina through a retrospective analysis of recent cases | 2016 | [Hashiguchi Y](https://www.ncbi.nlm.nih.gov/pubmed/?term=Hashiguchi%20Y%5BAuthor%5D&cauthor=true&cauthor_uid=26611809) | Universidad Central del Ecuador |
| 2 | First Evidence of a Hybrid of Leishmania (Viannia) braziliensis/L. (V.) peruviana DNA Detected from the Phlebotomine Sand Fly Lutzomyia tejadai in Peru. | 2016 | [Hashiguchi Y](https://www.ncbi.nlm.nih.gov/pubmed/?term=Hashiguchi%20Y%5BAuthor%5D&cauthor=true&cauthor_uid=26611809) | Universidad Central del Ecuador |
| 3 | A rapid molecular diagnosis of cutaneous leishmaniasis by colorimetric malachite green-loop-mediated isothermal amplification (LAMP) combined with an FTA card as a direct sampling tool | 2016 | [Hashiguchi Y](https://www.ncbi.nlm.nih.gov/pubmed/?term=Hashiguchi%20Y%5BAuthor%5D&cauthor=true&cauthor_uid=26611809) | Universidad Central del Ecuador |
| 4 | Insecticidal activity of modified turpentine oil in Culex quinquefasciatus and Aedes albopictus | 2015 | Tacorente J | Universidad Central del Ecuador |
| 5 | Capsaicin Fluidifies the Membrane and Localizes Itself near the Lipid–Water Interface | 2015 | Alessio A | Universidad Técnica Particular de Loja |
| 6 | DNA barcoding for identification of sand fly species (Diptera: Psychodidae) from leishmaniasis-endemic areas of Peru | 2015 | [Hashiguchi Y](https://www.ncbi.nlm.nih.gov/pubmed/?term=Hashiguchi%20Y%5BAuthor%5D&cauthor=true&cauthor_uid=26611809) | Universidad Central del Ecuador |
| 7 | Multilocus sequence typing approach for a broader range of species of Leishmania genus: describing parasite diversity in Argentina. | 2015 | [Hashiguchi Y](https://www.ncbi.nlm.nih.gov/pubmed/?term=Hashiguchi%20Y%5BAuthor%5D&cauthor=true&cauthor_uid=26611809) | Universidad Central del Ecuador |
| 8 | Population genetics of *Leishmania (Leishmania) major* DNA isolated from cutaneous leishmaniasis patients in Pakistan based on multilocus microsatellite typing | 2014 | [Hashiguchi Y](https://www.ncbi.nlm.nih.gov/pubmed/?term=Hashiguchi%20Y%5BAuthor%5D&cauthor=true&cauthor_uid=26611809) | Universidad Central del Ecuador |
| 9 | The isolation and molecular characterization of Leishmania spp. from patients with American tegumentary leishmaniasis in northwest Argentina | 2014 | [Hashiguchi Y](https://www.ncbi.nlm.nih.gov/pubmed/?term=Hashiguchi%20Y%5BAuthor%5D&cauthor=true&cauthor_uid=26611809) | Universidad Central del Ecuador |
| 10 | Effectiveness of the E2-Classical Swine Fever Virus Recombinant Vaccine Produced and Formulated within Whey from Genetically Transformed Goats | 2014 | Barrera M | Not specified |
| 11 | Angiotensin type 1a receptor-deficient mice develop diabetes-induced cardiac dysfunction, which is prevented by renin-angiotensin system inhibitors | 2013 | [Rachid Seqqat](https://www.ncbi.nlm.nih.gov/pubmed/?term=Seqqat%20R%5BAuthor%5D&cauthor=true&cauthor_uid=24215514) | Escuela Politécnica del Ejército |
| 12 | ELISA with Recombinant rKRP42 Antigen Using Urine Samples: A Tool for Predicting Clinical Visceral Leishmaniasis Cases and Its Outbreak | 2012 | [Hashiguchi Y](https://www.ncbi.nlm.nih.gov/pubmed/?term=Hashiguchi%20Y%5BAuthor%5D&cauthor=true&cauthor_uid=26611809) | Universidad Central del Ecuador |
| 13 | Polymorphism-specific PCR enhances the diagnostic performance of American tegumentary leishmaniasis and allows the rapid identification of *Leishmania* species from Argentina | 2012 | [Hashiguchi Y](https://www.ncbi.nlm.nih.gov/pubmed/?term=Hashiguchi%20Y%5BAuthor%5D&cauthor=true&cauthor_uid=26611809) | Universidad Central del Ecuador |


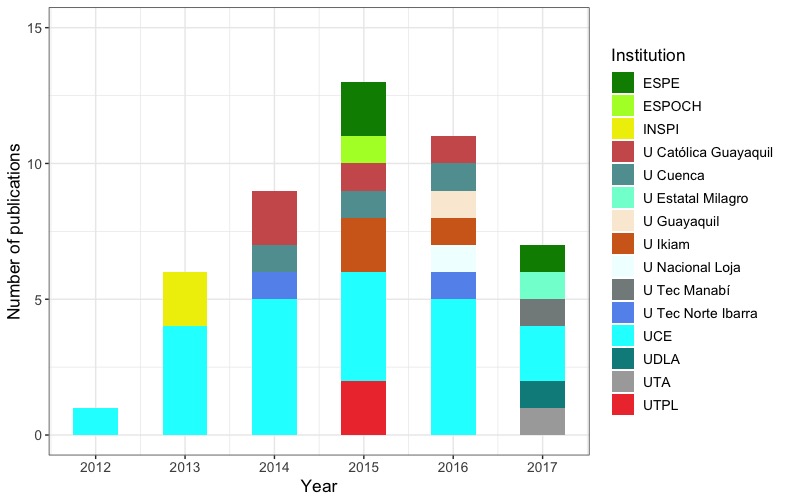


**Figure S1. Health science publications related to the Prometeo program according to institution affiliation.**

Data correspond to the analyzed period from 2009 to 2017 (n=47). Each square represents one Ecuadorian Higher Education Institution. Note. ESPE: Escuela Superior Politécnica del Ejercito, ESPOCH: Escuela Superior Politécnica de Chimborazo, INSPI: Instituto Nacional de Investigación en Salud Pública, UCE: Universidad Central del Ecuador, UDLA: Universidad de Las Américas, UTA: Universidad Ténica de Ambato, UTPL: Universidad Técnica Particular de Loja.


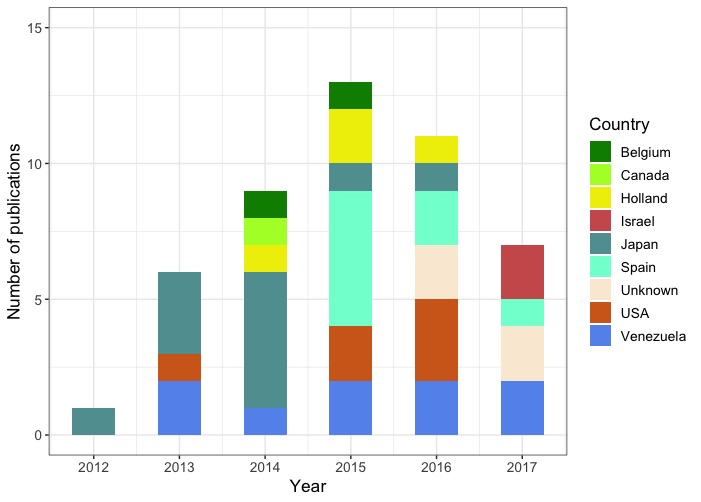


^a^

**Figure S2. Health science publications related to the Prometeo program by the country affiliation of the grant recipient.**

Data correspond to the analyzed period from 2009 to 2017 (n=47). Each square represents the home country from where the researcher applied to the Prometeo program.

^a^ Two papers did not specify the country or home institution affiliation of the Prometeo researcher.


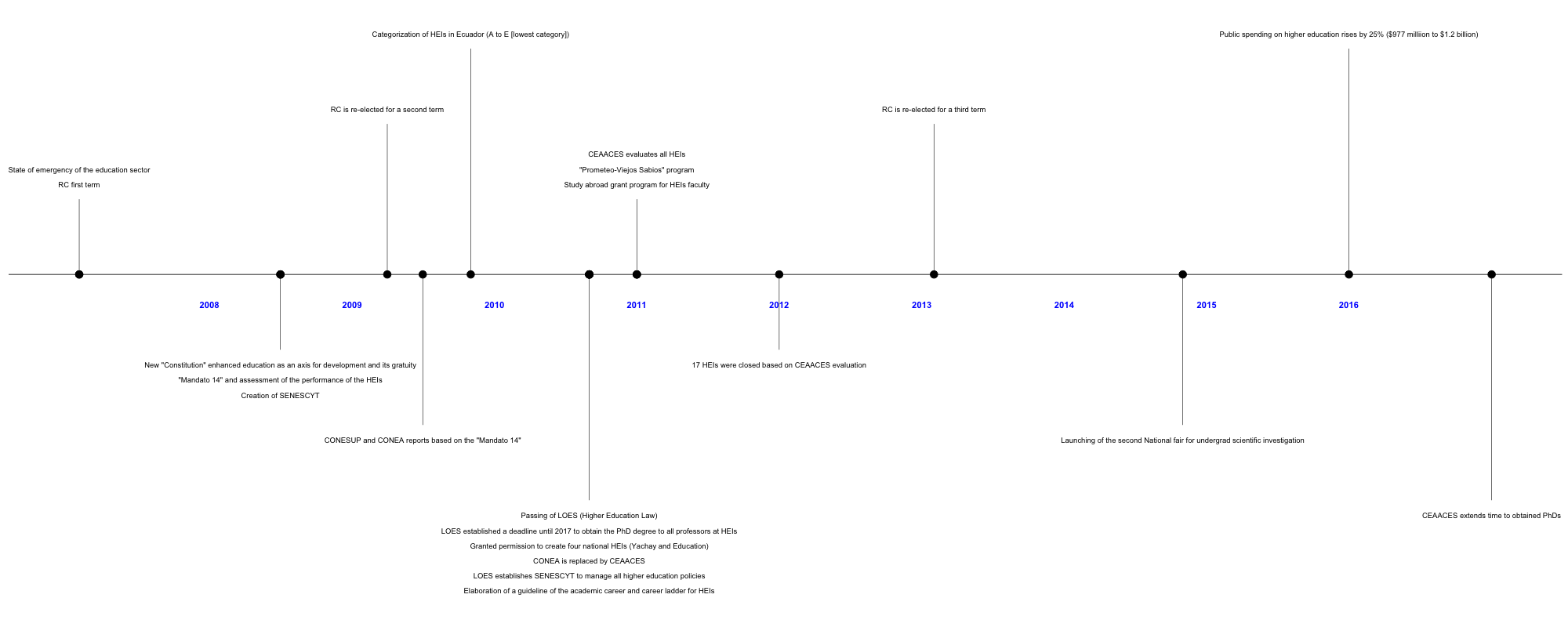


**Figure S3. Timeline of the milestones achieved in Ecuadorian higher education system during “Revolución ciudadana”, 2007-2017**

Note. RC: Rafael Correo; HEIs: higher education institutions; SENESCYT: Secretary of Higher Education, Science, Technology, and Innovation; CEAACES: Consejo de Evaluación, Acrediatación, y Aseguramiento de la Calidad de la Educación; CES: Higher Education Council; LOES: Higher Education Law; CONESUP: Consejo Nacional de Educación Superior; CONEA: Consejo de Evaluación, Acreditación y Aseguramiento de la Calidad de la Educación Superior del Ecuador
